# Extracellular Vesicles in Hematological Malignancies: From Biology to Therapy

**DOI:** 10.3390/ijms18061183

**Published:** 2017-06-02

**Authors:** Antonella Caivano, Francesco La Rocca, Ilaria Laurenzana, Stefania Trino, Luciana De Luca, Daniela Lamorte, Luigi Del Vecchio, Pellegrino Musto

**Affiliations:** 1 Laboratory of Preclinical and Translational Research, IRCCS-Referral Cancer Center of Basilicata (CROB), 858028 Rionero in Vulture, Italy; ilaria.laurenzana@crob.it (I.L.); stefania.trino@crob.it (S.T.); dr.luciana.deluca@gmail.com (L.D.L.); daniela.lamorte@crob.it (D.L.); 2 Laboratory of Clinical Research and Advanced Diagnostics, IRCCS-Referral Cancer Center of Basilicata (CROB), 85028 Rionero in Vulture, Italy; francesco.larocca@crob.it; 3 CEINGE-Biotecnologie Avanzate scarl, Federico II University, 80138 Naples, Italy; luigi.delvecchio@unina.it; 4 Department of Molecular Medicine and Medical Biotechnologies, Federico II University, 80138 Naples, Italy; 5 Scientific Direction, IRCCS-Referral Cancer Center of Basilicata (CROB), 85028 Rionero in Vulture, Italy; p.musto@tin.it

**Keywords:** extracellular vesicles, hematological malignancies, bone marrow microenvironment, drug resistance, biomarkers, therapeutic tools

## Abstract

Extracellular vesicles (EVs) are a heterogeneous group of particles, between 15 nanometers and 10 microns in diameter, released by almost all cell types in physiological and pathological conditions, including tumors. EVs have recently emerged as particularly interesting informative vehicles, so that they could be considered a true “cell biopsy”. Indeed, EV cargo, including proteins, lipids, and nucleic acids, generally reflects the nature and status of the origin cells. In some cases, EVs are enriched of peculiar molecular cargo, thus suggesting at least a degree of specific cellular packaging. EVs are identified as important and critical players in intercellular communications in short and long distance interplays. Here, we examine the physiological role of EVs and their activity in cross-talk between bone marrow microenvironment and neoplastic cells in hematological malignancies (HMs). In these diseases, HM EVs can modify tumor and bone marrow microenvironment, making the latter “stronger” in supporting malignancy, inducing drug resistance, and suppressing the immune system. Moreover, EVs are abundant in biologic fluids and protect their molecular cargo against degradation. For these and other “natural” characteristics, EVs could be potential biomarkers in a context of HM liquid biopsy and therapeutic tools. These aspects will be also analyzed in this review.

## 1. Introduction

Extracellular vesicles (EVs), a heterogeneous group of lipid bilayer particles between 15 nanometers and 10 microns in size, are released by almost all (normal and neoplastic) cell types [1]. They are classified, in increasing order of size, into exosomes (Exo) [2], microvesicles (also referred as ectosomes or microparticles; MVs) [3], small and large oncosomes [4,5], according to their biophysical properties (i.e., size, shape) and mechanism of biogenesis [6].

EVs contain a set of cytoplasmic and surface proteins, lipids [7], nucleic acids (DNA, mRNA, and small and long noncoding RNA) [8,9,10,11,12] and even cellular organelles, such as mitochondria [13]. The components of EVs, currently known, are summarized in some databases such as EVpedia, Vesiclepedia, and ExoCarta [14,15,16].

The EV cargo generally reflects the nature and status of the origin cell [17,18]. In some cases, EV cytoplasmic and surface content suggest a degree of specific cellular packaging into EVs [6,19].

The release of EVs, both in physiological and pathological conditions, including tumors, is influenced by microenvironment [20,21], oxygen tension [22], glucose [23] and intracellular Ca^2+^ concentrations [3], and the status of donor cells [17,18].

Once released from a given cell, EVs can reach target cells where they can remain stably associated with plasma membrane or can be internalized through diverse endocytic pathways with subsequent transfer of EV cargo into cytoplasm and nucleus of recipient cells [24,25,26,27,28]. In particular, Rappa et al. described spathasomes as a mechanism to transfer EV biomaterials into the nucleus of recipient cells [28]. Due to the ability to transfer their cargo to target cells, EVs can alter the composition and function of recipient cells and can even induce epigenetic changes [29,30,31,32]. EVs exert effects in a pleiotropic manner, by directly activating cell surface receptors via protein and lipid ligands, or by integrating their membrane content into the plasma membrane of their cellular targets [33,34].

EVs can be isolated from diverse body fluids [35], including blood [36], saliva [37], semen [38], seminal plasma [18], mother’s milk [39], synovial fluid [40], nasal secretions [18], urine, feces [18,41], amnion [42] , ascites [43], and cerebrospinal fluids [44]. In particular, we reported that serum MVs are elevated in patients with different hematological malignancies (HMs) when compared to healthy subjects and, more importantly, they exposed surface specific HM associated markers [36].

EVs have emerged as particularly interesting tumor information vehicles, such that they could be considered for “cell biopsies”. Therefore, EVs could be used as minimally invasive biomarkers and even as a therapeutic tool. Their role was extensively studied in solid tumors [45,46,47,48] whereas in HMs it is in the process of being defined [49,50,51].

The aim of this review is to discuss the recent advances in the field of EVs as actors in HMs, underlining the EV role in the tumor-microenvironment cross-talk, in drug resistance, as well as their potential use as biomarkers and therapeutic tools.

## 2. Physiological Role of Extracellular Vesicles (EVs)

EVs play important roles in intercellular cross-talk in both short and long distances [1,52]; in fact, they are involved in numerous physiological processes, including stem cell renewal and differentiation [53], tissue repair [31], immune surveillance [33], and blood coagulation [33,54]. For these reasons, they have been highly preserved through evolution [9,18].

Ratajczak et al. reported that MVs isolated from mouse embryonic stem cells (SCs) efficiently enhanced survival and expansion of murine hematopoietic progenitor cells (HPCs) [55]. A recent study showed that MVs from megakaryocytes (Mks) induced differentiation of hematopoietic stem and progenitor cells (HSPCs) towards the Mk lineage [56].

EVs seem to also play important roles in tissue engineering and regenerative activities, including liver, nervous, vascular, reproductive, and renal systems [57,58]. Indeed, EVs released from injured tissues, acting on SCs, promoted the release of “regenerative” MVs for tissue repair [59]. Gatti et al. reported that intravenous administration of EVs derived from human mesenchymal stem/stromal cells (MSCs) stimulated tubular cell proliferation and protected against acute kidney injury [31].

All the immune system cells, including macrophages, B, T, NK, and dendritic cells (DCs), can release EVs. A lot of evidence suggests that EVs have an important role in the regulation of immunity, acting both as immune stimulators or suppressors [6,18,52,60]. EVs acted as immune suppressors by enhancing the function of regulatory T cells (T-regs), inhibiting NK and CD8+ cell activity [33,61], and affecting monocyte differentiation [62]. Recently, Jong et al. reported that activated NK cells produced EVs that were active against cancer cells, delivering cytotoxic proteins, such as perforin and granzymes A and B [63]. Moreover, EVs from embryonic SCs affect DC maturation and both T cell proliferation and differentiation, thus potentially contributing to innate immune suppression [64]. Interestingly, healthy MSC-derived EVs also induced immunosuppressive effects on purified T, B, and NK cells from healthy donors [65].

One of the best characterized physiological roles of EVs is their capacity to enhance coagulation [18]. An inverse correlation between the EV number and their capacity to form both thrombin and thrombin-antithrombin complexes has been clearly demonstrated [18,66]. Furthermore, EVs found in saliva and urine of healthy humans exposed coagulant tissue factor (TF) and initiated TF/factor VII-mediated coagulation [29].

EVs are also involved in fertilization, pregnancy, and fetus/mother communication [18,67]. In amniotic fluid and in human breast milk, EVs regulate the immune response of fetus to maximize its survival during pregnancy and growth [18].

Finally, EVs participate in myelin formation, as well as in neurite outgrowth and neuronal survival of nervous system [68,69].

## 3. Role of EVs in the Malignancy-Microenvironment Cross-Talk

**Figure 1:**
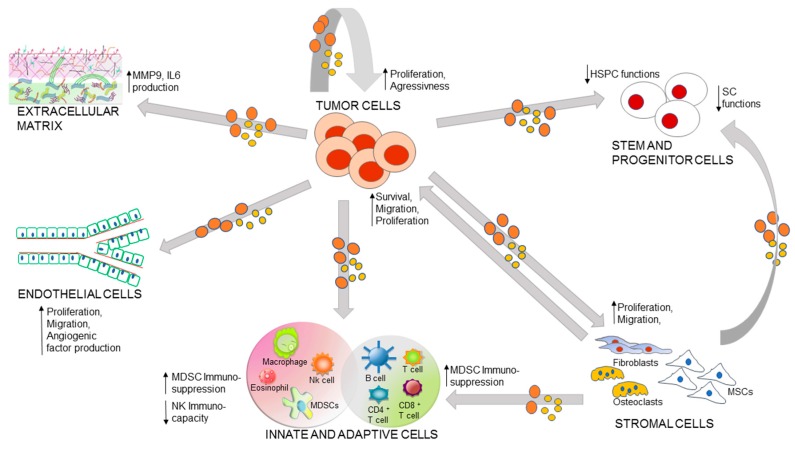
A summary drawing of the role of Extracellular vesicles (EVs) in bone marrow (BM) microenvironment of different hematological malignancies (HMs). BM cells release EVs which can act, through both autocrine and paracrine (gray arrows), on recipient cells. Tumor EVs can render themselves more aggressive and “stronger” stroma in supporting the tumors. In addition, neoplasm EVs interact with extracellular matrix to promote tumor invasion. Moreover, they enhance angiogenesis, inhibit the immune system, and induce a suppression of hematopoietic stem and progenitor cell (HSPC) functions and a stem cell (SC) malignant transformation. MMP9: Matrix metallopeptidase 9; IL6: interleukine 6; MDSC: Myeloid Derived Suppressor Cells; MSCs: mesenchymal stem/stromal cells; NK: natural killer cells.

The bone marrow (BM) hematopoietic niche consists of SCs, hematopoietic (both myeloid and lymphoid precursors), immune- and stromal cells (BMSCs); the latter includes vascular endothelial cells, pericytes, adipocytes, fibroblasts, osteoblasts/osteoclast, and MSCs, as well as extracellular matrix (ECM) [70,71]. All BM cell populations can significantly influence tumor microenvironment, via autocrine or paracrine mechanisms, through the secretion of a large variety of soluble factors, including EVs. In this context, EVs represent an important vehicle of growth factors, cytokines, enzymes, angiogenic molecules, and genetic materials that are able to reprogram and modify recipient cells [72]. The complex EV mediated bi-directional communications between tumor and BM microenvironment are summarized in Figure 1.

### 3.1. Autocrine Loop of Primary Tumor EVs

Several studies have suggested that, under different conditions, tumor cells produced EVs that modulated themselves, in an autocrine feedback loop [73,74,75], which is relevant for the concepts of tumor auto-sustaining and the increase of tumor aggressiveness.

It has been recently demonstrated that multiple myeloma (MM) EVs have a prominent role in promoting tumor plasma cell proliferation through both direct and indirect mechanisms. In particular, EVs derived from MM cells over-expressing CD147 (an extracellular matrix metalloproteinase inducer also known as basigin) enhanced neoplastic proliferation more than EVs released from CD147 downregulated cells, thus suggesting that CD147 is involved in EV induced MM proliferation [73].

Patel et al. reported that Exo derived from a pre-B acute lymphoblastic leukemia (ALL) cell line growing at high density induced a proliferative effect on the same non-growing low density cells, favoring their survival [76]. In addition, ALL-derived Exo also induced cell proliferation of leukemic and non-leukemic B cell lines in both an autocrine and paracrine manner [74].

Several leukemic cells, including erythromyeloblastoid, chronic myeloid leukemia (CML), and pre-B ALL cells, released their specific oncogenic fusion transcripts in MVs; interestingly, these EVs returned to leukemic cells with an autocrine loop [75]. In particular, CML-derived Exo promoted the proliferation and survival of CML cells, both in vitro and in vivo, by TGF-β1/TGF-β1 receptor engagement [77]. However, this EV autocrine loop did not work in a chronic lymphocytic leukemia (CLL) model [71]; a possible explanation could be that CLL cells did not express heparan sulphate (HS) receptors required for the EV uptake [78]. Further studies are needed to better define the autocrine processes involving EVs. Moreover, Paggetti et al. demonstrated that CLL-derived exosomes induce an inflammatory phenotype in endothelial and MSCs resembling the phenotype of cancer-associated fibroblasts. CLL-derived exosomes, in this way, create a favorable environment for promoting CLL progression [78]. Further studies are needed to better define the autocrine processes involving EVs.

### 3.2. EV Mediated Communication between Stem Cells and Malignancy

It is well established that the embryonic microenvironment is non-permissive for tumor development [79]. In this setting, Zhou et al. demonstrated in vitro and in vivo that Exo released by human embryonic SCs could reprogram cancer cells, inhibiting tumor growth and tumorigenicity. On the other hand, tumor cells can interact and modify the SCs through the EVs [79].

It has been demonstrated that leukemic SC derived MVs promote proliferation and migration and inhibit apoptosis of acute myeloid leukemia (AML) cells [80]. In addition, AML derived Exo suppressed residual HSPC functions by “decreasing” clonogenicity and by reprogramming stroma, respectively [81,82]. Recently, BM-AML-MVs promoted the survival of healthy hematopoietic stem cells (HSCs) without changing their immature phenotype and their ability to form colonies, but inducing leukemic-like functional characteristics, such as miR21 and miR29 over-expression [83]. Interestingly, researchers demonstrated an essential role of vacuolar protein sorting protein 33b (VPS33B) in Exo pathways of HSC and leukemia-initiating cells. In particular, the VPS33B deletion in an in vivo AML model led to impairment of both Exo maturation and secretion and delayed the onset of AML [84]. Finally, Koch et al. reported that diffuse large B-cell lymphomas possessed an infrastructure comprising hematopoietic stem cells (HSCs) and not HSCs. Exo mediated Wnt signaling modulated the transitions between clonogenic states [85].

### 3.3. Immuno-Cells and Tumor Cross-Talk

Depending on the type of cells from which they are released, EVs exert immune-modulatory activities, influencing both the activation and suppression of the immune response [86]. In particular, the release of EVs by neoplastic cells is one mechanism through which the antitumor immune response is suppressed or evaded [87].

For example, EVs derived from B and T cell lymphomas have been found to be enriched in major histocompatibility complex (MHC), T-cell receptor (TCR), Apo2 ligand (APO2L), Fas ligand (FasL), and natural-killer group-2 member-D (NKG2D), which had the ability to inhibit NK cell cytotoxicity, to promote T cell apoptosis, and to downregulate antigen (Ag) processing by Ag presenting cells [88,89,90].

In CLL, where NK cell dysfunction has been reported [91], the plasma soluble ligand BAG6 inactivated NK cell functions by binding their receptor NKp30. On the contrary, BAG6-positive Exo activated NK cells that could kill tumor cells [92]. Therefore, a possible explanation of CLL immune evasion could be a deregulated balance between BAG6-Exo and BAG6 soluble form.

Both MM cell and BMSC-Exo promoted the growth and enhanced the immunosuppressive activity of myeloid derived suppressor cells (MDSCs) in vitro and in MM xenograft [93,94]; moreover, MM-Exo reduced cytotoxic activity of NK cells against MM cells [95]. In addition, CD38-positive EVs released from MM cells could represent an MM strategy to escape immune system. In fact, ectoenzyme CD38 on MM EVs could generate an anergic immune system by converting nucleotides to adenosine, which is an immune-suppressor; in addition, CD38-positive EVs binding anti-CD38 mAb (Daratumumab) could be captured by FcR-positive cells such as NK, monocytes, and MDSCs [96,97]. The effects of EV internalization by immune cells are under investigation.

Exo from Burkitt’s lymphoma cell line (DG75), from latent membrane protein1 (LMP1)-transfected cell line (LMP-DG75) or from EBV (Epstein-Barr virus)-infected cell line (DG75-EBV) was used in order to mimic Exo produced during EBV infection or EBV-associated diseases. DG75 Exo were internalized by B cells in PBMCs, and led their proliferation and T cell independent class-switch recombination. Additionally, LMP1-DG75 Exo induced the differentiation of B cells into a plasmablast-like phenotype [98].

In Hodgkin lymphoma (HL), Hansen et al. reported that Hodgkin and Reed Stemberg (HRS) cells released CD30-positive EVs that mediate communication with supportive microenvironment. In particular, CD30+ EVs stimulated innate immune cells, such as healthy eosinophil-like EoL-1 cells and primary granulocytes to release Interleukin (IL)-8 [99].

TGFβ1 plays a significant role in leukemic Exo-mediated immune escape [100]. Exo from sera of AML patients containing membrane-associated TGFβ1 reduced the ability of NK cells to kill leukemic cells; furthermore, their presence in sera correlated to response to chemotherapy in AML [101]. TGFβ1 has also been found to be enriched in CML-Exo and treatment with TGF-β1 inhibitor significantly reduced Exo-stimulated cell proliferation and colony formation of CML cells [100].

### 3.4. EV Mediated Bi-Directional Communication between BM Stroma and Tumor

EVs participate in the cross-talk between malignant cells and BMSCs [93,102,103,104], exerting either an anti- or a pro-tumor growth effect, depending on cancer type and stage of disease [62,105].

Ghosh et al. found that CLL-MVs play an important role in the activation of CLL microenvironment in favor of disease progression [106]. In particular, CLL-MVs can activate the AKT signaling pathway in CLL-BMSCs by inducing the production of vascular endothelial growth factor (VEGF), a survival factor for CLL cells [107]. Moreover, CLL-Exo facilitate a transition of stromal cells towards cancer-associated fibroblast phenotype and promote cell migration and angiogenesis in vitro and in vivo [78,108].

Fei and colleagues demonstrated the protective effects of stroma versus ALL cells following the release of EVs contained Galectin-3 (GAL3) [109]. GAL3was internalized by ALL cells, where it stimulated transcription of endogenous GAL3 mRNA and was a protection toward drug treatment [109]. Also, ALL Exo could be captured from stromal cells, inducing a metabolic switch from oxidative phosphorylation to aerobic glycolysis [110].

The interaction between BMSCs and MM cells plays a key role in MM pathogenesis [93]. BMSC-derived Exo induced survival, proliferation, and migration of MM cells in an in vivo MM mouse model, while normal BM-MSC-Exo significantly reduced MM cell proliferation [102]. Interestingly, Exo from cells of MM patients, under hypoxia conditions, accelerate in vitro angiogenesis; this effect is due to miR135b, which targets the HIF1-dependent signaling pathway [111]. Notably, it has been reported that MM-MVs carrying multiple angiogenesis-related proteins enhanced angiogenesis by modulating the STAT3 pathway and that endothelial cells stimulated with MM-MVs secrete IL-6 and VEGF, two important factors for MM cell growth [112]. Moreover, Exo from MM cells and MM patient sera also interacted with osteoclasts (OCs), supporting their growth and migration and inducing a differentiation toward multinuclear OCs which eroded dentine discs, thus suggesting a possible role in the development of bone disease of MM patients [113]. Vardaki and colleagues firstly provided evidence that Bcl-xL, a caspase-3 substrate, was presented in the BM-derived fibroblast Exo and that the consequent cleaved Bcl-xL (clBcl-xL) was exposed on Exo surface. The clBcl-xL was necessary for EV uptake by both MM and aggressive lymphoma cells, driving their proliferation [114].

Exo derived from adult T-cell leukemia/lymphoma (ATL) cells transferred epigenetic regulators, such as miR21 and miR155, induced changes in cellular morphology, and promoted proliferation of human MSCs to support the creation of a favorable microenvironment for leukemia [115].

Horiguchi et al. demonstrated that EVs derived from leukemic cells were efficiently transferred into MSCs [116]. In particular, they found that EV miR-7977 derived from AML/myelodysplastic syndrome (MDS) CD34+ cells, was transferred into BM MSCs and reduced their ability to support CD34+ cells. Huan et al. studied the role of Exo in developing BM AML niche [117]. They reported that AML-Exo altered the proliferative and migratory responses of both BMSCs and hematopoietic progenitor cell lines, resulting in the re-programming of the microenvironment. As recently suggested by Muntion et al., MVs derived from MSCs of MDS patients strongly modify CD34+ cell properties, promoting their cell viability and clonogenic capacity and altering micro-RNA and gene expression profiling [118].

EVs released by MSCs from patients with myeloproliferative neoplasms (MPN) were found to be selectively enriched in miR155, and they induced an increase in granulocyte colony forming unit number from neoplastic CD34+ [119]. Moreover, Exo released by CML cells stimulate BMSCs to produce IL-8 which, in turn, promotes both in vitro and in vivo leukemic cell survival [104]. MVs containing “leukemic” transcripts from CML cells transferred these mRNA in healthy MSCs, increasing their proliferation [75]. In addition, CML-Exo were able to enhance angiogenesis in vitro; this effect was amplified when the Exo were released in hypoxic conditions [120]. BCR-ABL1-positive MVs from CML also induced malignant transformation of normal mononuclear cells through genomic instability via different mechanisms, which led to DNA breakage and recombination [121]. Interestingly, Gutkin et al. demonstrated that enzyme telomerase (hTERT) mRNA may be transferred, via Exo, from cancer cells, such as T-cell leukemia and CML cells, to hTERT negative fibroblasts. In these cells, hTERT mRNA was translated into an active form that transforms them into telomerase-positive cells [122]. Of interest, hTERT expression in hTERT negative cells affected their biological properties towards a tumor like-phenotype, characterized by extended or unlimited life span, prevention and reversal of senescence, resistance to DNA damage, and prevention of apoptosis [19,123].

### 3.5. EVs and Extracellular Matrix (ECM)

Purushothaman and colleagues revealed a new mechanism for Exo-mediated cross-talk within MM cells [124]. In particular, these authors demonstrated that surface heparan sulfate (sHS)-positive (sHS+) Exo were able to bind fibronectin (fibro), then act as ligand for sHS+ target cells. Specifically, the interaction between MM cells and fibro-sHS+ Exo induced the activation of p38 and pERK and the production of DKK1 and MMP9, two crucial factors for MM invasion. Moreover, anti-fibro antibodies (Abs) inhibited the invasion of endothelial cells mediated by MM Exo [124].

Redzic et al. demonstrated that EVs from multiple cancer cells stimulate the secretion of EMMPRIN, MMP9, and IL-6, important mediators of migration and inflammation, in human monocytic leukemia cells, thus suggesting a further manner in which EVs drive tumor progression [125].

Finally, in 3D Matrigel culture and in HL tissues, CD30+ EVs from HL cells predominantly stuck to long actin- and tubulin-based protrusions; this could be a further network to guide CD30+ EVs into the microenvironment towards distant cells, thereby easing their communication [99].

### 3.6. Malignancy-Microenvironment Cross-Talk and Drug Resistance

Drug resistance in cancer is a multi-factorial process due to intracellular mechanisms (e.g., gene alterations or epigenetic changes) and to interactions of cancer cells with the microenvironment, resulting in decreased drug accumulation, increased efflux, drug compartmentalization, and alterations of cellular pathways [126]. Emerging evidence has highlighted the role of EVs to confer drug resistance in cancer cells, for example, by sequestering drugs and/or by reducing their free concentration available to cells [127]. In this setting, EVs shed from donor drug-resistant to recipient drug-sensitive cells can transfer drug resistance through different modulators, such as drug-efflux pumps, miRNAs, and long noncoding RNAs [128].

Recently, Koch at al. demonstrated that lymphoma cell lines treated with anthracyclines, drugs currently used for the therapy of aggressive lymphoma, secreted anthracycline-containing Exo [129]. Moreover, the silencing of ABCA3 transporter using the cyclooxygenase inhibitor indomethacin suppressed the biogenesis of Exo, with a consequent increase in drug susceptibility of lymphoma cells in vitro and in vivo*.* Therefore, the authors suggested that the targeting of Exo biogenesis could provide a promising approach to overcome drug resistance and to enhance antitumor efficacy of anthracyclines [129].

EVs could exert a negative effect on the therapy efficacy in HMs. For example, BMSC-Exo inhibited bortezomib mediated cell apoptosis in MM cells [103]; likewise, GAL3 Exo from stromal cells have been reported to activate the NFkB pathway in ALL cells, inducing an anti-apoptosis effect and drug resistance [109].

CD20-positive Exo in CLL can bind anti-CD20 monoclonal Ab, Rituximab, reducing its free action on CLL cells [78,130]. Similarly, in B cell lymphoma mouse models, tumor cells evaded complement-mediated killing of immunotherapy through the action of lymphoma Exo, which binds complement [131].

In an AML setting, Viola et al. found that stromal Exo trafficking could be a candidate mechanism for extrinsic chemo-resistance which increases tyrosine kinase inhibitor resistance [132]. An AML cell line carrying FLT3 internal tandem duplication (ITD) mutation was exposed to Exo derived from normal or AML BMSCs. Both Exo types protected AML cells from cytarabine effects, while only AML-BMSC-Exo protected AML cells from FLT3 inhibitor treatment [132]. Recently, it has also been shown that AML cells resistant to apoptosis could modulate, via EVs, the expression profiling of apoptosis-related proteins of blasts sensitive to chemotherapy [133]. Finally, a multi-resistant AML cell line transferred its chemo resistance to sensitive promyelocytic leukemia cells through EVs [134]. These results indicate a first proof of concept that circulating AML-EVs could become potential biomarkers for therapy resistance. However, further studies are needed to define their clinical implications.

Interestingly, in another setting, EVs can enhance drug efficacy to kill both tumor cells and their supportive microenvironment. In HL, for example, the anti-CD30 Ab drug conjugate, Brentuximab Vedotin (SGN-35), bound CD30+ EVs released by HRS cells, forming SGN-35/CD30+ EV complex [135]. The authors reported that SGN-35 directly killed tumor cells (CD30+ cells). In addition, the tumor supportive microenvironment, including mast cells and eosinophils (CD30 negative cells), was damaged by SGN-35/CD30+ EV internalization [135].

## 4. Clinical Potential of EVs as Biomarkers and Therapeutics

EVs are abundant in biofluids, protect their molecular cargo against degradation, and may deliver genetic/proteic/lipidic signatures associated with specific phenotypes. They could be, therefore, considered a full-fledged form of “cell biopsy”, with several advantages in respect to circulating tumor cells, that are less present in the circulation, and in respect to cell-free circulating biomarkers, including proteins, microRNA, and others, which are susceptible to degradation and have short half-lives [136]. The aforementioned characteristics, together with the possibility “to engineer” EVs, make them attractive as new possible biomarkers and therapeutic tools in HMs.

### 4.1. EVs as Biomarkers

We have previously reported that serum MV count in CLL, non-Hodgkin’s lymphoma (NHL), Waldenstrom’s macroglobulinemia (WM), HL, MM, AML, MPN, and MDS patients are high compared to healthy subjects [36].

In CLL, our data are in agreement with those provided by Gosh et al. about the amount of CLL-EVs [106]. Moreover, we reported that total number of MVs in CLL patients positively correlates with advanced clinical stages, it is predictive for overall survival (OS), and, in patients with initial stages, it also correlates with time to treatment. Furthermore, serum CLL-MVs were preferentially positive for CD19 and CD37 [137]. In a different setting, Boysen et al. showed that plasma CD52+ MVs were preferentially released from CLL cells and that they can be a predictive biomarker of progression [107]. Recently, an antibody microarray (DotScan) of plasma CLL-EVs gave a profile of surface proteins. These EVs expressed moderate or high levels of CD5, CD19, CD31, CD44, CD55, CD62L, CD82, HLA-A, B, C, and HLA-DR and low levels of CD21, CD49c, and CD63 [138].

Regarding the EV genetic cargo, our and other data suggest that serum EV miR155 level is a promising prognostic/predictive biomarker in CLL, independent of clinical stage [139,140].

In HL, we demonstrated that HL MVs are positive for CD30, the phenotypic marker of HRS cells, and that patients with higher HL stage showed fewer CD30+ MV than low stage HL subjects [36]. Further studies will be performed to clarify this data. Van Eijndhoven et al. confirmed our results in terms of high levels of EVs in patients with respect to controls [141]. Moreover, we and others reported that plasma EV miRNA levels, including miR155, miR127, miR21, and let7, reflect the presence of the tumor and could be a diagnostic tool for therapy response and relapse monitoring in HL [139,141].

In WM, we showed, for the first time, higher levels of serum MVs and EV miR155 with respect to controls [36,139]. The miR155 amount also had a positive correlation trend with the International Prognostic Scoring System. Interestingly, Exo miRNA content may also differ at different phases of disease [142] and this could potentially be used as a marker of progression in patients with WM.

MM circulating EVs are enriched in CD38, CD138, CD44, superficial HSP70, and CD147 [36,73,143,144]. In these studies, the number of serum MVs CD38+ positively correlated with clinical international stage system, plasma CD138+ MVs were associated with disease phase and therapeutic response, and increased plasma CD147+ MVs were found at disease progression. Finally, Harshman et al. identified, by EV proteomic profiling, CD44 as a novel associated marker in MM [145].

In regard to circulating MM EV cargo, different authors have reported that MM EVs contained c-SRC, ZNF224, CCL2, IL-6, fibro and various miRNAs, including miR15 and 16 and miR135b [145,146]. In particular, Exo miR135b was found to enhance angiogenesis in MM bone marrow; on the contrary, its expression level in plasma Exo was significantly lower when compared to controls, thus suggesting a possible role in local BM rather than in circulation. Recently, Manier et al. indicated serum Exo derived microRNAs, let7b and miR18a as significant predictors of progression free survival (PFS) and OS in MM patients [147], supporting the possibility that circulating miRNA from Exo could be predictive of MM prognosis.

Very little information is currently available about the role of circulating EVs in MPNs, though we demonstrated that CD13+ MVs were higher in the serum of CML and primary myelofibrosis patients with respect to healthy subjects [36]. Regarding CML, a differential miRNA signature between K562 cell derived-Exo and K562 cells has also been found [148]; preliminary data indicated that plasma Exo miR215 could have a possible role in selecting CML patients who can discontinue imatinib therapy [149].

We also reported increased levels of circulating serum MVs (in particular of those positive for CD13, a “mature” myeloid surface marker) in MDS patients with respect to healthy controls. Interestingly, higher risk MDS showed the highest amount of CD13+ MVs, comparable with those detected in AML [36].

Circulating AML-EVs are enriched in CD34 [150], CD 117 [151], and CD13 [36]. Plasma AML Exo have a considerably greater amount of proteins with respect to controls [132]; in particular, protein content of Exo from AML cells resistant to chemotherapy could give relevant information about drug resistance [132]. Therefore, AML-Exo TGFβ1 levels could be a novel biomarker of response to therapy and could reflect the presence/absence of residual disease after therapy [101].

Exo from AML cells contain mRNA that are important for AML prognosis (FLT3-ITD, NPM1), for treatment, (FLT3-ITD, IGF-IR, CXCR4), and for behavior of leukemic niche (IGF-IR, CXCR4, MMP4) [117]. In regard to miRNA cargo in AML-Exo, we reported that EV miR155 was over-expressed in AML serum when compared to healthy subjects [139]. Its amount was directly correlated with white blood count cells and complex karyotypes and could be further investigated as a biomarker in AML patients. Interestingly, in an AML xenograft mouse model, Exo miR155 and other miRNAs were indicative of AML presence, thus suggesting their potential use for minimal residual disease detection [81,152].

Finally, in the allogeneic stem cell transplant (alloSCT) setting, EVs have been reported as potential biomarkers of acute graft versus host disease (GvHD). In particular, in MM patients after allograft, CD46 and CD25-positive EVs were associated with an increased risk of developing a GvHD, while CD31 and CD106-positive EVs were correlated with a reduced incidence of this complication [153]. Furthermore, plasma EVs from HMs patients undergoing alloSCT contains myeloid, erythroid, and megakaryocyte lineage specific transcripts. Interestingly, the presence of these EV mRNAs seems to precede and predict the recovery of white blood cells, reticulocyte, and platelet count in blood, thus providing a novel potential biomarker of allogeneic stem cells engraftment [154].

All the information about EVs as biomarkers in HM is summarized in Table 1.

### 4.2. EVs for Therapy

EVs are currently under investigation as attractive and possible tools to integrate within novel therapeutic strategies in immunotherapy, as therapeutic targets, and as mechanisms of drug delivery [155].

#### 4.2.1. Immunotherapy

EVs may act not only as Ag carriers, but also as modulators of direct and indirect Ag presentation [64]. For instance, specific Ags carried by tumor EVs can be taken up and processed by DCs; at this point, DCs can prime tumor-specific cytotoxic T-lymphocytes to initiate the immune response [155,156,157]. Indeed, some studies have indicated the possibility of using tumor-derived Exo as vaccines in lymphoma because they have been shown to be immunogenic and effective for DC vaccination. For example, Yao et al. showed that CML Exo-pulsed DC induced strong cytotoxic antileukemic immune responses as well as protective immunity against leukemia cells in vitro and in vivo [158,159]. In experiments reported by Shen et al., HSP70+ Exo were derived from a human acute promyelocytic leukemia cell line. These HSP70+ Exo were pulsed on DC and showed to be much more effective in inducing leukemia-specific cytotoxic T lymphocytes than non-pulsed DC [160]. Exo carrying proteins important for antigen presentation were shown to elicit T cell cytotoxic activity in vitro and in vivo and to induce Th1-type antibody responses via activation of B cells [161]. Thus, combining several immune-stimulatory signals in EVs could generate EV-based vaccines that might induce potent innate and adaptive immune responses [162]. To date, the possibility of using EVs as a tool in cell free-vaccines is under consideration.

Another potential immunotherapeutic application of EVs is their use as agents to restore NK cell activity in some HMs. In particular, in CLL, where NK cell dysfunction has been reported [91], BAG6-positive Exo represents a trigger for NK cytotoxicity [92].

In the scenario of EVs as an immune-therapeutic tool, it is important to underline the role of MSC-EVs. By recapitulating both immune-modulator and cytoprotective features of parental cells, they have therapeutic/protective effects in both tissue repair and regeneration [163,164]. Moreover, MSC-EVs could be used to improve stem cell engraftment in alloSCT. In this setting, we recently demonstrated that BM-MSC-EVs are able to modify the phenotype of CD34-positive hematopoietic stem cells and increase their migration from peripheral blood to BM in an in vivo model [165]. Finally, after alloSCT, MSC-EVs have been also employed to treat GvHD refractory in therapy [166].

#### 4.2.2. EVs as Therapeutic Target

The importance of EVs in intercellular cross-talk of tumors renders them as potential therapeutic targets. Different approaches can be applied to stop EV-mediated communications: (i) reducing the amount of EVs by interfering with their biogenesis and release; (ii) blocking EV uptake by target cells; (iii) interfering with their path in target cells; and (iv) removing EVs from the circulation.

Exo biogenesis could be attenuated by small-molecule inhibitors of enzymes or proteins such as ALIX involved in Exo formation [167]. Exo secretion can also be regulated by increasing intracellular Ca^2+^ levels (as observed in K562 CML cells) [168] or by the use of dimethyl amiloride (DMA), an inhibitor of Na^+^/Ca^2+^ exchange [169]. In particular, DMA was shown to reduce Exo secretion in mice bearing EL4 lymphoma. It induced inhibition of tumor growth accompanied by suppression of MDSC activity [169]. Targeting Exo biogenesis could also provide a promising approach to overcome drug resistance and to enhance anti-lymphoma efficacy of anthracyclines and anthracenediones [129].

Uptake of EVs could be targeted and/or attenuated by blocking surface adhesion molecules, such as phosphatidylserine, ICAM1, sHS, proteoglycans, or other receptors important for EV internalization [170,171]. The targeting of intracellular signaling pathways activated by Exo or oncogenes delivered to recipient cells using inhibitory RNAs is also being explored [172].

There are no data regarding the depletion of circulating EVs in HM. On the contrary, depletion of cancer-derived Exo was applied to suppress cancer metastasis. Recently, Nishida et al. demonstrated that the administration of human-specific anti-CD9 and anti-CD63 Abs, binding these Ags on Exo surface, suppressed metastasis to the lungs, lymph nodes, and thoracic cavity in a human breast cancer xenograft mouse model [173].

#### 4.2.3. Therapeutic Drug Delivery

EVs can be exploited as drug delivery tools thanks to a series of interesting “natural” features:

(1) An architecture that protects their cargo from nucleases and proteases;
(2) Size (nanometer) and specific composition (lipid bilayer) that minimize recognition by the mononuclear phagocyte system [174];
(3) Low immunogenicity (patient self-derived EVs reduced the adaptive immune system activation);
(4) Specific lipids and proteins, such as CD55 and CD59, which stabilize EVs in bodily fluids [52,175], stimulate membrane fusion between cells;
(5) Tropism to target specific cells and tissues [176].

EVs have been evaluated as a drug delivery vehicle for diverse therapeutic cargos, including both small molecules (e.g., doxorubicin, curcumin, etc.) and macromolecules (i.e., RNA, DNA, and proteins)**.** There are different EV loading strategies, such as ex vivo extracellular EV loading and in vitro intracellular loading during EV biogenesis [177].

Several drugs, like doxorubicin, withferin A, and celastrol, delivered by Exo and compared to free drugs administration, improved anti-tumor effects in in vivo models, such as colon adenocarcinoma and breast cancer [178]. Furthermore, mixing curcumin with Exo improved the bioavailability and anti-inflammatory efficacy of this drug in a model of LPS(Lipopolysaccharide)-induced septic shock [179].

Regarding to macromolecule delivery, different miRNA and silencing RNA were loaded in EVs from different cells [3,180]. For example, Lunavat et al. demonstrated that Exo delivered cMyc silencing RNA reduced cMyc transcript and induced apoptosis in mouse lymphoma cells [180].

#### 4.2.4. Storage Conditions and Bio-Distribution of EVs

Nowadays, there are no protocols for EV manipulation for therapeutically purposes. EV purification and storage processes, in fact, need to be standardized in order to make them more reproducible and safe and to preserve EV functional properties. It has been demonstrated that purification methods of EVs can dramatically affect their integrity, influencing their subsequent bio-distribution in vivo [181,182]. Furthermore, the natural matrix of EVs might interfere with their integrity. EVs need to be stored as a “pure” pool for therapeutic applications [182]. A large number of solvents and buffers such as water, Phosphate Buffered Saline (PBS), Dimethyl sulfoxide (DMSO), and glycerol have been tested for EV storage; among them, the last two induced a full or partial lyses of EVs [183] while PBS was widely used but it interfered with EV quantification based on single particle detection [184]. Recently, Lener et al. suggested storing EVs in isotonic buffers to prevent pH shifts also during the freezing and thawing procedures [182]. In addition, concerning the storage temperature, it seems that EVs integrity is more preserved at −80 and −20 °C than at −190 or at 4 °C [183,185]. However, in consideration of the wide spectrum of potential EV-based applications, ad hoc purification and storage protocols need to be developed.

## 5. Conclusions

In HM, EVs are important intercellular “communicators”. They are involved in the tumor auto-sustaining, in making the stroma “stronger” in supporting malignancy, in inducing tumor drug resistance, and in immune system suppression. In addition, EVs could be potential biomarkers in the context of HM liquid biopsy and are interesting therapeutic tools thanks to their abundance in biologic fluids, to their natural capacity to protect molecular cargo, and, finally, to the possibility of engineering them.

Overall, several questions such as the lack of EV population specific identity and cell origin, the insufficient information about their biology and activity in disease and health, the lack of standardized isolation methods, and the need an implementation of EVs as drug delivery platform, still remain open**.** Recently, an EV-TRACK platform was created to recruit all EV biological and technical information [186] in order to render more transparent the field of EVs.

## Figures and Tables

**Table 1:** EVS as biomarkers in HMs.

Disease	Biofluids	Ev Type	Tumor Biomarkers	References
**CLL**	Serum, plasma	MVs	CD19, CD37, CD52	[36,107,137]
	Serum	MVs and Exo	miR155	[139,140]
**HL**	Serum	MVs	CD30	[36]
	Serum, plasma	MVs and Exo	miR155, miR127, miR21, miR24	[139,141]
**WM**	Serum	MVs and Exo	miR155	[139,140]
**MM**	Serum, plasma, cell medium	MVs and Exo	CD38, CD138, CD147, CD44, cSRC, ZNF224, CCL2, IL6	[36,73,101,143,144]
	Plasma	MVs and Exo	miR15, miR16, miR135, let-7b, miR181	[145,147]
**MM patients after allograft**	Serum	EVs	CD46, CD31, CD106, CD25	[153]
**CML**	Serum	MVs	CD13	[36]
	Plasma	Exo	miR 215	[149]
**PMF**	Serum	MVs	CD13	[36]
**MDS**	Serum	MVs	CD13	[36]
**AML**	Serum, plasma, cell medium	MVs and Exo	CD34, CD117, CD33, CD13, TGFβ1	[36,132,150,151]
	Serum, plasma	EVs	miR155	[139,152]
	Cell medium	Exo	FLT3-ITD, NPM1, IGF-IR, CXCR4 mRNAs	[117]
**HM patients after allograft**	Plasma	EVs	DEFA3, HBB, ITGA2B, ITGB3 mRNAs	[154]

CLL = chronic lymphocytic leukemia; HL = Hodgkin lymphoma; WM = Waldenstrom’s macroglobulinemia; MM = multiple myeloma; CML = chronic myeloid leukemia; PMF = Primary myelofibrosis; MDS = myelodysplastic syndrome; AML = acute myeloid leukemia; FLT3-ITD = FMS-like tyrosine Kinase 3 with internal tandem duplication; NPM1 = Nucleophosmin 1; IGF-IR = Insulin-like growth factor 1receptor; CXCR4 = Chemokine receptor-4; DEFA3 = Defensin,a 3, neutrophil-specific; HBB = Hemoglobin b; ITGA2B = Integrin a 2; ITGB3 = Integrin b 3, platelet glycoprotein IIIA.

## Abbreviations

ABCA3	ATP-binding cassette member A3
Ag	Antigen
GvHD	Graft versus Host Disease
ALIX	ALG-2-interacting protein X
ALL	Acute Lymphoblastic Leukemia
AML	Acute Myeloid Leukemia
APO2L	Apo2 Ligand
BL	Adult T-Cell Leukemia/Lymphoma Burkitt’s Lymphoma
BM	Bone Marrow
BMSCs	Bone Marrow Stromal Cells
c-SRC	Proto-oncogene tyrosine-protein kinase Src
CCL2	C-C Motif Chemokine Ligand 2
clBcl-xL	Cleaved Bcl-xL
CLL	Chronic Lymphocytic
CML	Chronic Myeloid Leukemia
CXCR4	Chemokine receptor type 4
DCs	Dendritic Cells
DKK1	Dickkopf-1
DMA	Dimethyl Amiloride
ECM	Extracellular Matrix
EMMPRIN	Extracellular Matrix Metalloproteinase Inducer
EVs	Extracellular Vesicles
Exo	Exosomes
FasL	Fas Ligand
fibro	Fibronectin
FLT3	fms-Like Tyrosine Kinase 3
GAL3	Galectin-3
HIF-1	Hypoxia-Inducible Factor 1
HL	Hodgkin Lymphoma
HM	Hematological Malignancy
HPCs	Hematopoietic Progenitor Cells
HPS-70	Heat Shock Protein 70
HRS	Hodgkin and Reed Stemberg
HS	Heparan Sulphate
HSCs	Hematopoietic Stem Cells
HSCT	Hematopoietic Stem Cell Transplantation
HSPCs	Hematopoietic Stem and Progenitor Cells
hTERT	Enzyme Telomerase
ICAM1	Intercellular Adhesion Molecule-1
IGF-IR	Insulin-like Growth Factor-I Receptor
IL	Interleukin
ITD	Internal Tandem Duplication
MDS	Myelodysplastic Syndrome
MDSCs	Myeloid Derived Suppressor Cells
MHC	Major Histocompatibility Complex
Mks	Megakaryocytes
MM	Multiple Myeloma
MMP4	Matrix metallopeptidase 4
MMP9	Matrix metallopeptidase 9
MPN	Myeloproliferative Neoplasm
MSCs	Mesenchymal Stem/Stromal Cells
MVs	Microvesicles
NFkB	Nuclear Factor kappaB
NHL	Non-Hodgkin’s Lymphoma
NKG2D	Natural-Killer Group-2 Member-D
NPM1	Nucleophosmin 1
OCs	Osteoclasts
OV	Overall Survival
PFS	Progression Free Survival
SCs	Stem Cells
sHS	Surface Heparan Sulphate
TCR	T-cell Receptor
TF	Tissue Factor
TGF	Tumor Growth Factor
Th1	T helper 1
VEGF	Vascular Endothelial Growth Factor
WM	Waldenstrom’s Macroglobulinemia
ZNF224	Zinc Finger Protein 224
